# Exercise Training Regulates Cortical GPCR-Mediated Signaling Networks Through cAMP, Calcium, and Neuroactive Ligand–Receptor Interaction Pathways in Diabetic–Obese Rats: An In Silico Study

**DOI:** 10.3390/ijms27125602

**Published:** 2026-06-21

**Authors:** Yin-Yu Chiang, Michael Anekson Widjaya, Shin-Da Lee

**Affiliations:** 1 PhD Program for Health Science and Industry, China Medical University, Taichung 406040, Taiwan; u112310801@cmu.edu.tw; 2 Graduate Institute of Biomedical Sciences, College of Medicine, China Medical University, Taichung 40402, Taiwan; michaelanekson@gmail.com; 3 PhD Program in Healthcare Science, Department of Physical Therapy, China Medical University, Taichung 406040, Taiwan

**Keywords:** calcium signaling, cAMP, cerebral cortex, diabetes, G protein-coupled receptors, neuroactive ligand–receptor interaction, obesity, physical training, transcriptomics

## Abstract

Exercise-induced regulation of cortical GPCR pathways in diabetic obesity remains unclear. This study aimed to investigate exercise-associated GPCR-related transcriptomic pathway changes in the cerebral cortex of diabetic-obese rats. Cerebral cortical samples from male Zucker Fatty Diabetes Mellitus (ZFDM) rats subjected to a 12-week swimming program were examined using RNA sequencing, functional enrichment, GSOAP clustering, and STRING-based protein–protein interaction (PPI) analysis. Exercise training reduced fasting glucose and body weight. RNA sequencing identified 817 exercise-responsive transcripts (403 upregulated and 414 downregulated; *p* < 0.05), including 48 associated with GPCR signaling. Results showed that these 48 genes mapped to three major GPCR-related networks: cAMP signaling, with increased Adcyap1r1, Gipr, Tshr, and Vipr2 and decreased Vip, Chrm1, Gabbr2, and Sst; calcium signaling, with increased Ntsr1 and Trhr and decreased Chrm1; and neuroactive ligand–receptor interaction, with increased Trh, Trhr, and Crh and decreased Opr-related transcripts. These findings provide hypothesis-generating evidence for interpreting cortical GPCR-related transcriptomic pathway associations in diabetic-obese conditions.

## 1. Introduction

G protein-coupled receptors (GPCRs) regulate insulin secretion, appetite control, and inter-organ metabolic communication. Altered GPCR expression has been documented in obesity and diabetes, including transcriptomic differences in human islets from lean and obese donors, suggesting that metabolic stress modifies GPCR-dependent regulatory pathways [1,2,3]. GPCR dysregulation has also been reported in adipose tissue under obese conditions [4], supporting the notion that GPCR signaling is sensitive to metabolic state and may contribute to impaired glucose homeostasis in diabetes. Beyond peripheral tissues, GPCR signaling in the central nervous system contributes to feeding behavior, neuroendocrine regulation, and metabolic responses. Disruption of specific GPCR subtypes has been linked to altered body weight control and reduced physical activity in obese models [5]. Additional studies indicate that GPCR-related signaling may influence neurodegenerative processes and cognitive function [6,7]. The cerebral cortex thus emerges as a metabolically responsive region in which GPCR regulation may shift during obesity, although its transcriptional changes under metabolic stress remain insufficiently defined.

Given that GPCR signaling is sensitive to metabolic state, interventions targeting metabolic dysfunction may influence these pathways. Exercise produces well-established metabolic benefits, including improved glucose control, reduced inflammation, and enhanced oxidative enzyme capacity in obesity and diabetes [8]. Exercise training also induces molecular and structural adaptations in the brain, as shown in studies of neurotrophic, metabolic, and behavioral changes [9,10]. These adaptations suggest that cortical signaling networks are regulated by exercise training. However, despite advances in metabolic and neuronal profiling of GPCR, the cerebral cortical GPCR response to exercise training under diabetic–obese conditions remains poorly understood. Given this gap, we examined cerebral cortical GPCR-related transcriptional regulation to exercise training in diabetic obese rats using RNA sequencing and pathway-based analyses after swimming training. GPCR-associated genes identified from Reactome annotation were further analyzed through protein–protein interaction networks to determine major downstream signaling pathways. We sought to identify exercise-regulated GPCR signaling networks in the diabetic–obese cortex and to construct pathway-specific models of transcriptional regulation.

## 2. Results

### 2.1. Identification of Exercise-Responsive Transcripts in the Diabetic–Obese Cortex

Physiological outcomes of the exercise training are summarized in Supplementary Table S7. Animal allocation for physiological assessment and RNA-seq analysis is summarized in Supplementary Table S5. The 12-week swimming program produced clear metabolic improvements in diabetic–obese rats. Body weight was 10.4% lower in the OB-EX group compared with sedentary OB controls (487 ± 18 g vs. 543 ± 22 g, *p* < 0.05), and fasting glucose showed a pronounced reduction of 40.2% (185 ± 19 mg/dL vs. 310 ± 25 mg/dL, *p* < 0.05). Systolic blood pressure in OB-EX decreased modestly (−8.1%) from 138 ± 5 to 127 ± 4 mmHg (*p* < 0.05) compared with the OB group. Soleus citrate synthase activity—a marker of mitochondrial oxidative capacity of exercising muscle—was 84.3% higher in exercise-trained rats (0.42 ± 0.05 vs. 0.23 ± 0.04 U/mg protein, *p* < 0.01). These physiological changes confirm the effectiveness of the exercise training and establish the metabolic context for subsequent transcriptomic analyses.

**Figure 1:**
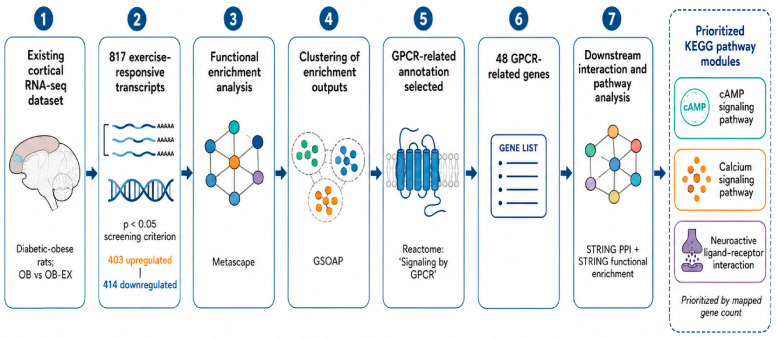
In silico workflow for GPCR-related clustering and pathway analysis. Processed bulk cortical RNA-seq data from exercise-trained and sedentary diabetic-obese rats were screened to identify 817 exercise-responsive transcripts using *p* < 0.05 as an exploratory criterion. Functional enrichment was performed using Metascape, and enrichment outputs were clustered using GSOAP. The Reactome “Signaling by GPCR” annotation was selected for downstream STRING PPI and STRING functional enrichment analysis. The prioritized KEGG pathway modules were ranked by mapped gene count. Abbreviations: GPCR, G protein-coupled receptor; RNA-seq, RNA sequencing; PPI, protein–protein interaction; KEGG, Kyoto Encyclopedia of Genes and Genomes.

A total of 817 exercise-responsive transcripts were identified between OB-EX and OB groups under the *p*-value < 0.05. This set included 403 upregulated and 414 downregulated transcripts (Supplementary Table S1). These transcripts were analyzed using GSOAP to perform pathway enrichment and clustering based on the Reactome database. Through this approach, G protein-coupled receptor (GPCR) signaling was identified as an enriched category. A total of 48 genes were annotated to the GPCR-related set derived from the Reactome/STRING workflow. Representative genes from this set are shown in Supplementary Table S2. These GPCR-associated genes were then re-analyzed using the STRING database to examine protein–protein interaction structures and related downstream pathways. From this analysis, three major pathways containing the largest number of GPCR-associated genes—cAMP signaling, calcium signaling, and neuroactive ligand–receptor interaction—were selected for detailed interpretation (Figure 1). This prioritization strategy was predefined to focus interpretation on the most gene-rich GPCR-linked modules identified through STRING enrichment analysis while retaining biological relevance to exercise-associated metabolic and neuroendocrine regulation. The overall workflow is summarized in Supplementary Table S6.

### 2.2. Reactome-Based Functional Clustering Identified GPCR Signaling as an Exercise-Associated Pathway

**Figure 2:**
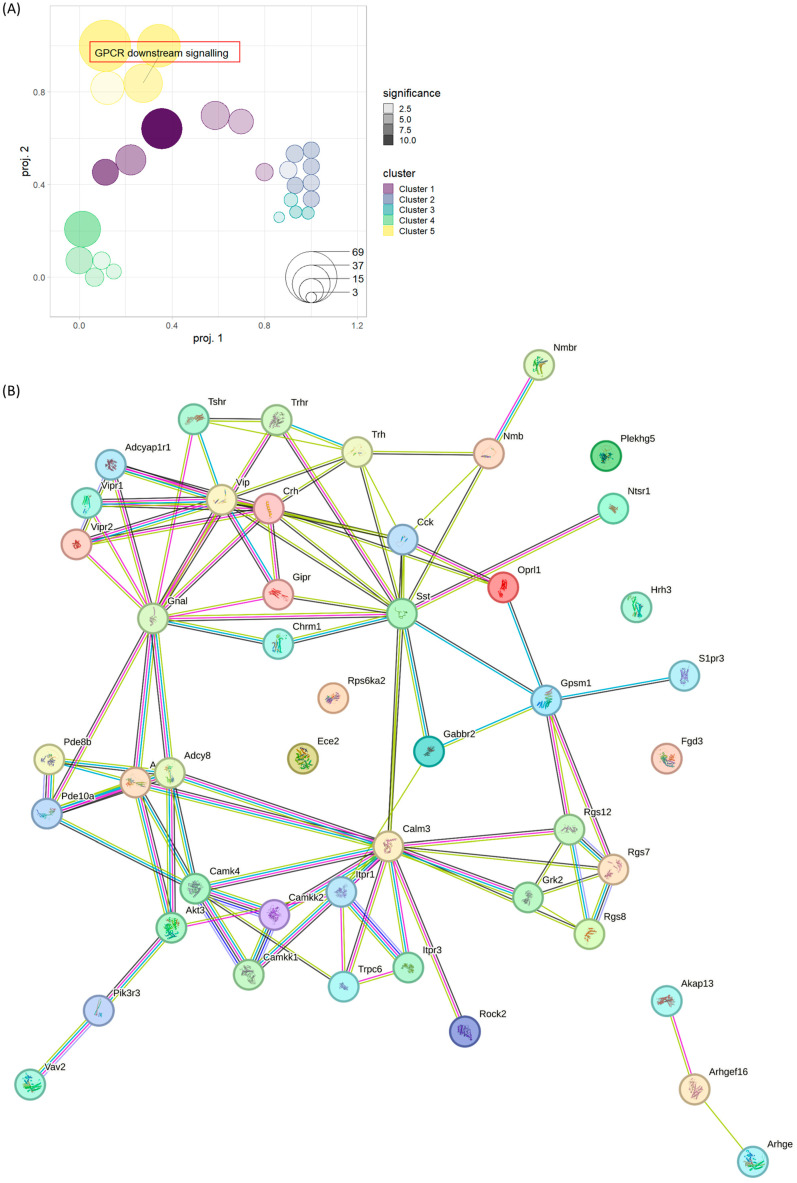
GSOAP clustering and STRING PPI analysis of GPCR-related genes. (**A**) GSOAP clustering/enrichment output showing the GPCR-related cluster, with Reactome “Signaling by GPCR” highlighted. (**B**) STRING PPI network of GPCR-related genes used for downstream KEGG pathway prioritization. Node colors are used for visualization within this network only and do not indicate fixed pathway membership, fold change, or direct color matching across different figure panels.

Pathway clustering using the GSOAP analytical framework revealed several Reactome-based functional categories after exercise training. Among these clusters, signaling by G protein-coupled receptors (GPCRs) was identified as an enriched pathway after exercise training, appearing within the same cluster as cAMP signaling, GPCR downstream signaling, and neuroactive ligand–receptor interactions (Figure 2A). This cluster contained a set of genes that demonstrated consistent transcriptional changes after exercise training. To further resolve the structure of this GPCR-associated gene set after exercise training, protein–protein interaction (PPI) analysis was performed using the STRING database. The PPI network exhibited extensive interconnections among GPCR-related genes, indicating that these genes participate in multiple interacting signaling modules rather than isolated pathways (Figure 2B). This network-based organization provided the basis for selecting downstream pathways for further analysis.

### 2.3. STRING-Based Prioritization of the Top Three GPCR-Related Pathway Modules

**Figure 3:**
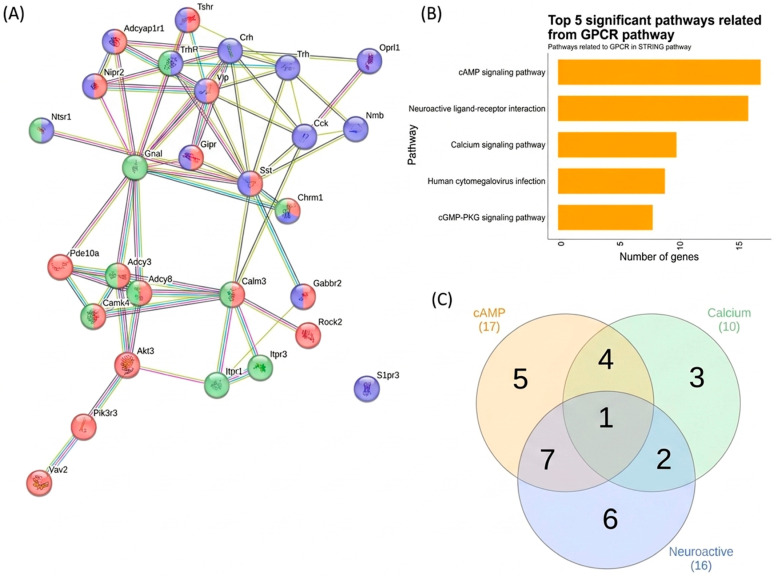
Top three GPCR-related KEGG pathway modules and gene overlap. (**A**) STRING PPI network of genes mapped to the top-three prioritized KEGG pathway modules. (**B**) STRING functional enrichment analysis showing the top three KEGG pathway modules ranked by mapped GPCR-related gene count. (**C**) Gene overlap among the three prioritized KEGG pathway modules. Chrm1 was identified as the gene shared across cAMP signaling, calcium signaling, and neuroactive ligand–receptor interaction. Detailed pathway membership, mapped genes, and enrichment summaries are provided in Supplementary Tables S2–S4. Abbreviations: GPCR, G protein-coupled receptor; PPI, protein–protein interaction; cAMP, cyclic adenosine monophosphate; KEGG, Kyoto Encyclopedia of Genes and Genomes. Node colors are used for visualization within the top-three-module network and should not be interpreted as exclusive pathway assignment. The three modules were prioritized according to mapped gene count derived from STRING functional enrichment analysis.

Re-analysis of the GPCR-associated genes using the STRING database identified three downstream KEGG pathway modules with the highest number of related genes: cAMP signaling, calcium signaling, and neuroactive ligand–receptor interaction (Figure 3A; Supplementary Table S4). These pathways represented the major signaling modules connected to the GPCR cluster. Protein–protein interaction mapping showed that genes from these three pathways were interconnected, forming a composite network rather than three isolated modules after exercise training (Figure 3A,B). Several genes appeared across more than one pathway, indicating areas of overlap within the GPCR-related gene regulation. Among these, a small subset contributed to two pathways, and Chrm1 was identified as the transcript shared across all three prioritized modules (Supplementary Table S3).

To further clarify the directionality of GPCR-related transcriptional changes after exercise training, genes mapped to the three prioritized pathway modules were classified according to their expression changes in OB-EX rats compared with OB controls. In the cAMP signaling module, Adcyap1r1, Gipr, Tshr, and Vipr2 were increased, whereas Vip, Chrm1, Gabbr2, and Sst were decreased after exercise training. In the calcium signaling module, Ntsr1 and Trhr were increased, whereas Chrm1 was decreased. In the neuroactive ligand–receptor interaction module, Trh, Trhr, and Crh were increased, whereas Opr-related genes were decreased. These directional patterns indicate that exercise training was associated with both pathway-specific and overlapping GPCR-related transcriptomic changes in the diabetic-obese cortex.

## 3. Discussion

Exercise training produced measurable physiological improvements in diabetic–obese rats, including reductions in fasting glucose and body weight and an increase in citrate synthase activity of exercising muscle. RNA sequencing identified 817 exercise-responsive genes, and pathway clustering highlighted GPCR signaling as a Reactome category. Subsequent STRING analysis of GPCR-associated genes revealed three principal downstream pathways: cAMP signaling, calcium signaling, and neuroactive ligand–receptor interaction. These pathways shared overlapping components and showed distinct transcriptional adjustments in response to exercise. Together, these findings summarize the major GPCR-related transcriptional responses observed in the cortex under diabetic–obese conditions. These findings should be interpreted as transcript-level and pathway-level associations, not as evidence of protein-level GPCR signaling or functional receptor activity.

### 3.1. Exercise-Associated Modulation of the cAMP Signaling Pathway

**Figure 4:**
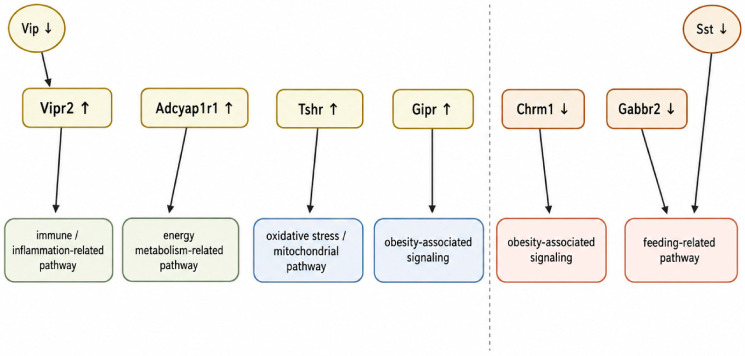
cAMP signaling-related hypothesis-generating model. The schematic summarizes putative transcript-level associations among GPCR-related transcripts mapped to the cAMP signaling module. Arrows indicate transcript direction in OB-EX compared with OB. The model does not indicate confirmed protein signaling, receptor activity, cell-type localization, or functional outcomes. Abbreviations: cAMP, cyclic adenosine monophosphate; GPCR, G protein-coupled receptor.

Several GPCR-associated genes linked to the cAMP pathway were altered following exercise training. Increased transcripts included Adcyap1r1, Gipr, Tshr, and Vipr2. Adcyap1r1 encodes the PAC1 receptor and has been linked to cAMP-related signaling in metabolic and neural contexts [11]. Gipr has recognized roles in metabolic regulation and incretin-mediated responses [12]. Cerebral cortical Tshr expression also increased after exercise training, consistent with reports describing Tshr involvement in oxidative and metabolic regulation in diabetic models [13]. Increased cerebral cortical Vipr2 after exercise training is consistent with evidence describing VIP/VPAC receptors in immune and metabolic modulation [14]. In contrast, expression of cerebral cortical Vip, Chrm1, Gabbr2, and Sst decreased after exercising training. Reduced cerebral cortical Vip expression after exercise training is consistent with reports linking VIP alterations to metabolic and inflammatory states [15]. Decreased cerebral cortical Chrm1 expression after exercise training is consistent with reports describing cholinergic alterations in cortical and metabolic conditions [16]. Lower cerebral cortical Gabbr2 expression after exercise training is linked with evidence that GABA-related pathways influence feeding and obesity models [17,18]. Reduced cerebral cortical Sst expression after exercise training is also compatible with reports describing somatostatin’s influence on food intake and weight outcomes [19]. These gene-level shifts suggest transcript-level associations within GPCR-linked cAMP signaling (Figure 4), and the model remains hypothesis-generating.

### 3.2. Exercise Training-Associated Modulation of Calcium Signaling

**Figure 5:**
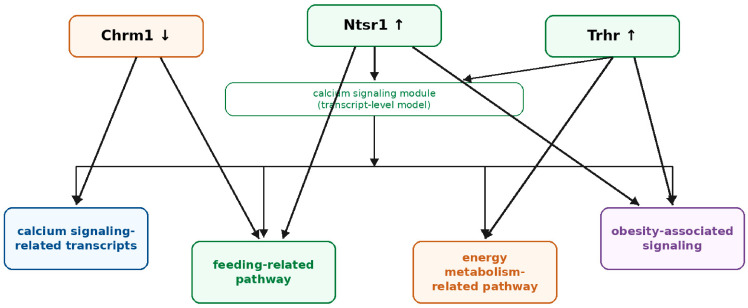
Calcium signaling-related hypothesis-generating model. The schematic summarizes putative transcript-level associations among GPCR-related transcripts mapped to the calcium signaling module. Arrows indicate transcript direction in OB-EX compared with OB. The model does not imply cell-type localization, calcium flux, protein-level signaling, or confirmed functional calcium signaling changes. Abbreviations: GPCR, G protein-coupled receptor.

Exercise training was associated with alterations in cerebral cortical GPCR-associated genes related to calcium signaling. Increased cerebral cortical transcripts included Ntsr1 and Trhr. Ntsr1, encoding neurotensin receptor 1, is linked to dopaminergic and feeding-related regulation in metabolic models [20]. Increased cerebral cortical Trhr expression after exercise training is linked with observations showing that brain TRH systems are sensitive to metabolic changes [21]. In contrast, cerebral cortical Chrm1 expression decreased after training, linked with reports that altered muscarinic receptor activity in the cortex has been documented in metabolic and diabetic states [16]. Calcium signaling has recognized roles in obesity-associated metabolic regulation [22], and the current cerebral cortical transcriptomic changes suggest GPCR- and calcium-associated pathway-level associations after exercise training (Figure 5).

### 3.3. Exercise Training-Associated Changes in Neuroactive Ligand–Receptor Interaction

**Figure 6:**
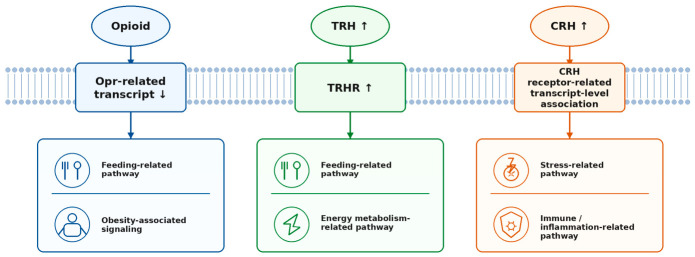
Neuroactive ligand–receptor interaction hypothesis-generating model. The schematic summarizes putative transcript-level associations among GPCR-related transcripts mapped to the neuroactive ligand–receptor interaction module. Arrows indicate transcript direction in OB-EX compared with OB. The model does not indicate confirmed receptor activation, protein-level signaling, cell-type localization, or behavioral outcomes. Abbreviations: GPCR, G protein-coupled receptor.

Within the neuroactive ligand–receptor pathway, exercise training increased cerebral cortical expression of Trh, Trhr, and Crh. Prior work demonstrates that Trh systems respond rapidly to metabolic conditions [21]. Similarly, altered Crh expression has been described in rodent models predisposed to obesity [23]. In contrast, Opr expression decreased. Opioid signaling influences feeding and weight outcomes in obese Zucker rats [24] and nociceptin receptor antagonism decreases high-fat diet-driven overeating [25]. These patterns suggest an exercise-associated transcript-level shift across several cerebral cortical GPCR-linked neuroactive ligand–receptor interaction systems related to feeding-related and energy metabolism-related pathways (Figure 6).

Beyond the pathway-specific findings, the three cerebral cortical GPCR-related modulations demonstrated a coherent pattern of coordinated transcriptional adjustments. Several cerebral cortical genes regulated by exercise training appeared across more than one pathway, indicating that exercise training did not operate through isolated signaling routes but rather through an interconnected regulatory network. Among these overlapping elements, cerebral cortical Chrm1 emerged as the only transcript shared across all three prioritized pathway modules, linking cAMP signaling, calcium-associated cascades, and neuroactive ligand–receptor interaction. This convergence suggests that Chrm1-related transcriptomic overlap may represent a pathway-level convergence point in the diabetic-obese cortex.

Other overlapping genes, including Trhr, Vip, Adcyap1r1, Crh, and Ntsr1, connected pairs of pathways, further supporting network-level rather than pathway-specific modulation after exercise training. These shared genes spanned functions related to peptide hormone signaling, cholinergic tone, calcium-related pathways, and stress-appetite regulation. Together, the overlap structure derived from the STRING network analysis highlights that the cerebral cortical GPCR-associated transcriptional response to exercise training is characterized by both pathway-specific adjustments and shared regulatory components, forming an integrated transcriptomic framework rather than confirmed mechanisms.

**Figure 7:**
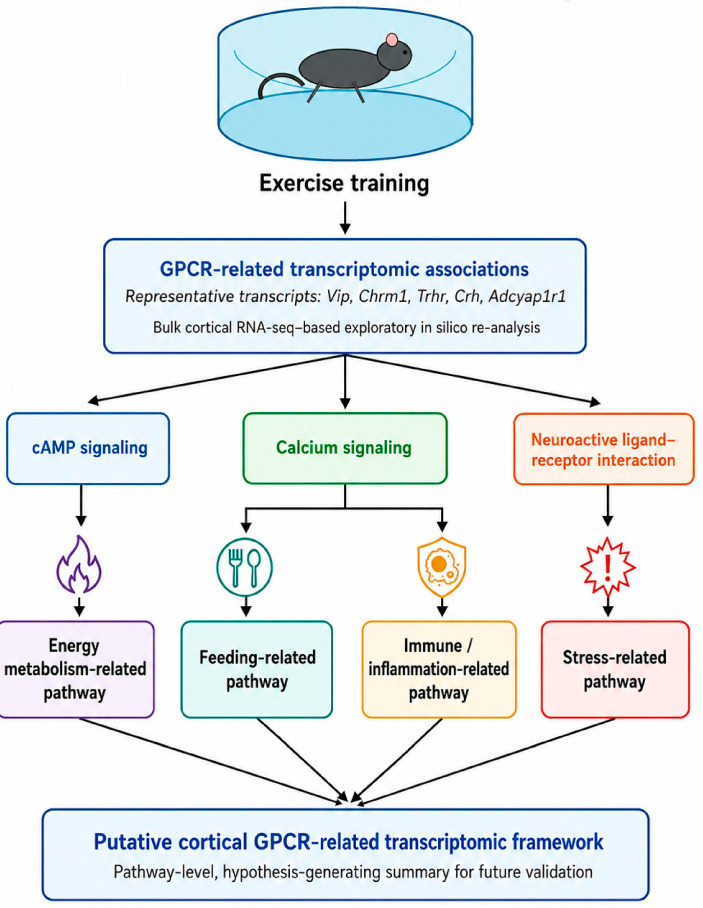
Integrated hypothesis-generating model of exercise-associated GPCR-related transcriptomic pathway associations. The schematic summarizes the putative cortical GPCR-related transcriptomic framework identified from bulk cortical RNA-seq and pathway-level re-analysis. The model is hypothesis-generating and does not indicate confirmed mechanistic or functional outcomes. Abbreviations: GPCR, G protein-coupled receptor; Vip, vasoactive intestinal peptide; Chrm1, cholinergic receptor muscarinic 1; Trhr, thyrotropin-releasing hormone receptor; Crh, corticotropin-releasing hormone.

Overall, the integrated model shown in Figure 7 summarizes the major cerebral cortical GPCR-related transcriptomic pathway associations after exercise training. Across analytical steps, cerebral cortical cAMP signaling, calcium signaling, and neuroactive ligand–receptor interaction consistently emerged as downstream pathways associated with GPCR-related transcriptional changes. Each pathway contained distinct and overlapping components, reflecting both specific and shared transcriptional effects. Although functional consequences cannot be inferred from transcript-level data alone, the model provides a descriptive, hypothesis-generating overview of GPCR-related transcriptomic adjustments that accompany exercise training in the diabetic–obese cortex.

### 3.4. Study Limitations and Future Directions

Several limitations should be noted. The cerebral cortical transcriptomic analysis was based on a limited sample size, which may restrict the detection of modest expression changes. The study assessed mRNA levels only, and no protein or functional assays were performed to confirm downstream effects. Pathway interpretation relied on bioinformatic prediction and network classification. Bulk cortical RNA-seq also prevents cell-type-specific inference, and the observed associations cannot be assigned to neurons, glia, vascular cells, or other cortical cell types. Although physiological outcomes and body weight were measured at the whole-animal and tissue levels after exercise training, no additional protein levels or functional validation were conducted to verify the downstream consequences. Processed gene-level expression outputs and pathway-analysis datasets can be made available from the corresponding author upon reasonable request and with appropriate data-sharing agreements. In addition, the analysis focused on cerebral cortical GPCR-related pathways as defined by Reactome annotations; other regulatory pathways not captured by the Reactome-defined GPCR annotation may have been overlooked. Future studies combining transcriptomic, protein-level, and functional validation will be required to determine whether these cerebral cortical GPCR-related transcript changes contribute to diabetic- or obesity-related signaling or behavioral consequences.

## 4. Materials and Methods

### 4.1. Animal Model and Experimental Design

Male Zucker Fatty Diabetes Mellitus (ZFDM) rats were used as a polygenic model of type 2 diabetes with obesity. Animals were maintained at 22 ± 2 °C under a 12 h light/dark cycle with free access to food and water. After acclimation, rats were randomly assigned to three groups (*n* = 10 per group): lean sedentary (LN), obese sedentary (OB), and obese exercise-trained (OB-EX). The animal cohort has also been described in a related cortical pathway analysis [26]. The present study performed a GPCR-focused re-analysis of our own previously collected diabetic-obese exercise RNA-seq dataset. For transcriptomic analysis, cerebral cortical samples from four animals in the OB and OB-EX groups were selected. Animal allocation for physiological measurements and RNA-seq analysis is summarized in Supplementary Table S5, and available physiological outcomes are summarized in Supplementary Table S7. All procedures complied with institutional and national animal care guidelines and were approved by the China Medical University IACUC (CMUIACUC-104-183-N).

### 4.2. Exercise Training Protocol

The OB-EX group underwent a 12-week swimming protocol adapted from earlier studies [27]. Rats swam 15 min/day during weeks 1–2, 20 min/day during week 3, and 30 min/day from weeks 4–12. Swimming training was performed five days per week in a temperature-controlled pool (60 × 90 × 50 cm, 35 °C). After each session, animals were dried gently with towels and warm air. All animals were euthanized 48 h after the final session to avoid acute exercise effects on molecular measurements.

### 4.3. Tissue Collection and RNA Isolation

Approximately 50 mg of cortical tissue was homogenized in 500 μL TRIzol reagent (Thermo Fisher Scientific, Waltham, MA, USA) containing stainless-steel beads (1:1:2–3 ratio of tissue: TRIzol:beads). Homogenization was performed using a TissueLyser II (Qiagen, Hilden, Germany) for two 2 min cycles at 20 Hz. Proteinase K (Worthington Biochemical Corp., Lakewood, NJ, USA) was added to minimize protein contamination. Phase separation was achieved using 1-bromo-3-chloropropane, followed by RNA precipitation with ethanol. The RNA was purified using the miRNeasy Mini Kit (Qiagen, Hilden, Germany) following the manufacturer’s instructions. RNA integrity was assessed using the Fragment Analyzer 5200 (Agilent, Santa Clara, CA, USA), and RNA concentration was measured using NanoDrop spectrophotometry (Thermo Scientific, Waltham, MA, USA).

### 4.4. RNA-Seq Library Preparation and Sequencing

Sequencing libraries were prepared from purified RNA using the TruSeq Stranded mRNA Library Prep Kit (Illumina, San Diego, CA, USA), following the manufacturer’s protocol. Poly-A mRNA was isolated from 1 μg of total RNA using oligo(dT)-coupled magnetic beads and fragmented at elevated temperature. First-strand cDNA synthesis was performed using reverse transcriptase and random primers, followed by second-strand cDNA synthesis, 3′ end adenylation, adaptor ligation, PCR enrichment, and purification using the AMPure XP system (Beckman Coulter, Beverly, MA, USA). Library quality was assessed using the Qsep400 System (Bioptic Inc., New Taipei City, Taiwan), and library concentration was measured using a Qubit 2.0 Fluorometer (Thermo Scientific, Waltham, MA, USA). Qualified libraries were sequenced on an Illumina NovaSeq platform to generate 150 bp paired-end reads by Genomics, BioSci & Tech Co. (New Taipei City, Taiwan).

### 4.5. Functional Enrichment, GSOAP Clustering, and STRING-Based PPI Analysis

Processed gene-level fragments per kilobase of transcript per million mapped reads (FPKM)-derived expression outputs from the bulk cortical RNA-seq dataset were used for the present GPCR-focused re-analysis. Downregulated transcripts were defined as postFC < 1, whereas upregulated transcripts were defined as postFC > 1, where postFC represents the fold-change ratio between the OB-EX and OB groups. Accordingly, all downstream analyses were interpreted as exploratory transcriptomic and pathway-level analyses based on the available processed gene-level output.

Exercise-responsive transcripts were screened from the processed gene-level FPKM-derived output using *p* < 0.05 as the screening criterion. The 817 exercise-responsive transcripts were then analyzed using Metascape [28], followed by GSOAP clustering [29], Reactome GPCR annotation, and STRING-based PPI and functional enrichment analyses. The enrichment outputs included Reactome and KEGG functional terms [30,31]. Genes annotated to the Reactome “Signaling by GPCR” term were analyzed using STRING [32] to construct protein–protein interaction (PPI) networks and to summarize downstream pathway modules. GPCR-linked pathways were ranked according to the number of mapped genes identified by STRING functional enrichment analysis. The three highest-ranking pathways (cAMP signaling, calcium signaling, and neuroactive ligand–receptor interaction) were selected for detailed interpretation because they represented the most gene-rich GPCR-associated modules and showed direct biological relevance to metabolic and neuroendocrine regulation in diabetic-obese conditions. Gene membership, pathway overlap, enrichment summaries, sample allocation, and workflow details are summarized in Supplementary Tables S1–S6. Physiological outcomes are reported separately in Supplementary Table S7.

## 5. Conclusions

This study demonstrates that exercise training is associated with cerebral cortical GPCR-related transcriptomic pathway changes in diabetic–obese rats. Among 817 exercise-responsive transcripts, GPCR-associated transcripts converged on three downstream modules—cAMP signaling, calcium signaling, and neuroactive ligand–receptor interaction—with both distinct pathway-specific patterns and shared network components. These findings outline the primary GPCR-related transcriptomic directions associated with exercise and provide a hypothesis-generating framework for future mechanistic investigations into cortical GPCR regulation under metabolic stress.

## Data Availability

The processed FPKM-derived screening output, GPCR-related pathway membership tables, STRING/KEGG enrichment summary, sample allocation, workflow summary, and physiological outcome summary are provided in Supplementary Tables S1–S7. Additional processed gene-level expression data and pathway-analysis files are available from the corresponding author upon reasonable request and with appropriate data-sharing agreements. Raw FASTQ files were not reprocessed in the present GPCR-focused re-analysis.

## Supplementary Materials

The following supporting information can be downloaded at https://www.mdpi.com/article/10.3390/ijms27125602/s1.
